# Insulin-Like Growth Factor 1 Predicts Post-Load Hypoglycemia following Bariatric Surgery: A Prospective Cohort Study

**DOI:** 10.1371/journal.pone.0094613

**Published:** 2014-04-15

**Authors:** Bianca K. Itariu, Maximilian Zeyda, Gerhard Prager, Thomas M. Stulnig

**Affiliations:** 1 Division of Endocrinology and Metabolism, Department of Medicine III, Medical University Vienna, Vienna, Austria; 2 Christian Doppler Laboratory for Cardio-Metabolic Immunotherapy, Medical University Vienna, Vienna, Austria; 3 Department of Surgery, Medical University of Vienna, Vienna, Austria; Queen's University Belfast, United Kingdom

## Abstract

Postprandial hypoglycemia is a complication following gastric bypass surgery, which frequently remains undetected. Severe hypoglycemic episodes, however, put patients at risk, e.g., for syncope. A major cause of hypoglycemia following gastric bypass is hyperinsulinemic nesidioblastosis. Since pancreatic islets in nesidioblastosis overexpress insulin-like growth factor 1 (IGF-1) receptor α and administration of recombinant IGF-1 provokes hypoglycemia, our main objective was to investigate the occurrence of post-load hypoglycemia one year after bariatric surgery and its relation to pre- and post-operative IGF-1 serum concentrations. We evaluated metabolic parameters including 2 h 75 g oral glucose tolerance test (OGTT) and measured IGF-1 serum concentration in thirty-six non-diabetic patients (29 f/7 m), aged 41.3±2.0 y with a median (IQR) BMI of 30.9 kg/m^2^ (27.5–34.3 kg/m^2^), who underwent elective bariatric surgery (predominantly gastric bypass, 83%) at our hospital. Post-load hypoglycemia as defined by a 2 h glucose concentration <60 mg/dl was detected in 50% of patients. Serum insulin and C-peptide concentration during the OGTT and HOMA-IR (homeostatic model assessment–insulin resistance) were similar in hypoglycemic and euglycemic patients. Strikingly, pre- and post-operative serum IGF-1 concentrations were significantly higher in hypoglycemic patients (p = 0.012 and p = 0.007 respectively). IGF-1 serum concentration before surgery negatively correlated with 2 h glucose concentration during the OGTT (rho = −0.58, p = 0.0003). Finally, IGF-1 serum concentrations before and after surgery significantly predicted post-load hypoglycemia with odds ratios of 1.28 (95%CI:1.03–1.55, p = 0.029) and 1.18 (95%CI:1.03–1.33, p = 0.015), respectively, for each 10 ng/ml increment. IGF-1 serum concentration could be a valuable biomarker to identify patients at risk for hypoglycemia following bariatric surgery independently of a diagnostic OGTT. Thus, IGF-1 testing could help to prevent a significant complication of gastric bypass surgery.

## Introduction

Bariatric surgery is currently the most effective treatment to achieve and maintain clinically significant long-term weight-loss in severely obese subjects [1]. The mortality rate from disease-related causes decreases in surgically treated patients, but the rate of non-disease deaths, caused by accidents, poisoning of undetermined intent or suicide is twice as high in postoperative patients compared to matched controls [2]. Postprandial, severe and recurrent hypoglycemia is a late complication of Roux-en-Y gastric bypass surgery (RYGB) [3] and could contribute to the post-operative increase in mortality unrelated to disease. Its occurrence is increasingly reported in recent bariatric surgery literature, with symptomatic or asymptomatic forms prevailing to a similar extent [4]–[6]. The published prevalence of hypoglycemia following bariatric surgery varies greatly depending on the applied definition with a range of 0.2% for patients requiring hospitalisation for severe hypoglycemia to 72% for reactive hypoglycemia in a glucose tolerance test [7], [8]. Postoperative hypoglycemia seems to be caused by post-prandial hyperinsulinemia, accompanied or not by pancreatic nesidioblastosis [9]–[11], or altered incretin release [12]–[14], even though other causes cannot be ruled out [9]. Hyperinsulinemic hypoglycemia has been described decades ago as a complication of gastrectomy and can lead to life-threatening neuroglycopenia [15]. Therapeutic approaches to prevent hypoglycemia following RYGB are not standardized and comprise dietary interventions, drugs, such as acarbose, diazoxide or verapamil as well as invasive procedures such as subtotal or total pancreatectomy [16], [17]. In order to prevent post-prandial hypoglycemia, pre-operative individual risk assessment would be imperative. However, factors predicting the onset of post-load hypoglycemia following bariatric surgery are widely unknown.

Insulin-like growth factor-1 (IGF-1) is a hormone with structural and functional similarities to insulin, whose main functions are regulation of somatic growth, proliferation and apoptosis. IGF-1 enhances insulin sensitivity and lowers glycaemia by stimulating glucose transport in muscle and adipose tissue via signalling mechanisms linked to insulin receptor activation [18]. It also enhances glucose uptake into several cells including osteoblasts by binding to the IGF-1 receptor [19]. Some glucose-related effects may be mediated through the IGF-2 and insulin receptors, although the binding occurs with low affinity. Furthermore IGF-1 promotes bone formation, protein synthesis, neuronal survival and also myelin synthesis [20]. During periods of food deprivation IGF-1 can reverse the negative nitrogen balance and inhibit protein catabolism in muscle [20]. Higher IGF-1 serum concentrations are associated with a higher risk of breast cancer in women and prostate cancer in men [21], [22]. IGF-1 concentration is directly related to insulin sensitivity, irrespective of confounders such as age, BMI, WHR or glucose tolerance status, but its association to insulin resistance seems U shaped [23], [24]. Bariatric surgery seems to have little effect on circulating IGF-1 concentrations [25], [26]. Recombinant IGF-1 is used as a long-term therapy in patients suffering from Laron Syndrome. Due to its insulin-like action, the most common side-effect to exogenous IGF-1 is hypoglycemia, which occurs in a dose-dependent manner [27].

Insulin-like growth factor 1 (IGF-1) receptor α is overexpressed in pancreatic islets in nesidioblastosis and administration of recombinant IGF-1 causes hypoglycemia [27], [28]. Given the direct effect of IGF-1 on glucose metabolism we hypothesised that IGF-1 is an indicator for the occurrence of post-prandial hypoglycemia in patients who underwent bariatric surgery. Consequently, high IGF-1 serum levels would be associated with hypoglycemia before and after bariatric surgery independent of insulin secretion. Therefore, we aimed to investigate the occurrence of post-load hypoglycemia by a 2 h OGTT and to assess the utility of IGF-1 serum levels before and after bariatric surgery as a biomarker for post-load hypoglycemia. Our data identified IGF-1 as a novel predicting factor for the occurrence of post-prandial hypoglycemia following bariatric surgery.

## Subjects and Methods

### Ethics statement

The study was conducted in compliance with the Declaration of Helsinki and Good Clinical Practice guidelines at the Department of Medicine III, Medical University of Vienna, and has been previously approved by the Ethics Committee of the Medical University of Vienna (EK Nr. 963/2009). All subjects provided written informed consent.

### Subjects

In this prospective study, we performed longitudinal measurements of metabolic and routine parameters in non-diabetic patients shortly before and approximately one year after the patients underwent elective bariatric surgery (mean follow up time 14±1.9 months). Surgeries were performed at the Department of Surgery of the Medical University of Vienna. We invited patients >18 years of age, who participated in our previous studies [29], [30] and from which we collected preoperative anthropometric and metabolic data (n = 55) to participate in this study. Patients were excluded in case of acute illness within the last two weeks, diagnosed type 2 diabetes, acquired immunodeficiency (HIV infection, AIDS), significant liver disease, severe or untreated cardiovascular, renal, pulmonary disease, active malignant disease, etc. Pregnancy or breast feeding were also among the exclusion criteria. Of the 55 patients we aimed to recruit, six patients did not respond to our invitation, five patients refused to participate or did not show up at the scheduled visit, one patient underwent surgery due to complications related to the initial procedure, one patient had died, and four patients were pregnant. We thus recruited 36 patients. Of these, 30 patients underwent RYGB, 3 patients underwent gastric banding and 3 patients underwent sleeve gastrectomy. None of the subjects were on oral antidiabetics of any kind, insulin, or corticosteroids. Before surgery 6 patients were on diuretics but treatment discontinued after surgery. One patient was on antipsychotic mediation before and after surgery. Antipsychotics can influence blood glucose concentration but the medication was continuously administered at both time points, before and after surgery. All samples and data were collected at the Clinical Research Unit of the Division of Endocrinology and Metabolism of the Medical University of Vienna. We advised patients to fast overnight before the examination. Preoperatively and at the follow-up visit, we performed anthropometric and hemodynamic measurements (body mass index (BMI), waist and hip circumference, systolic and diastolic blood pressure), blood sampling for routine laboratory analyses and a standardized 75 g 2 h oral glucose tolerance test (OGTT). Due to technical reasons we failed to perform the OGTT in one patient. After short venous stasis and venipuncture, blood was collected into evacuated vials and the samples were transferred to the Department of Laboratory Medicine of the Medical University of Vienna, where serum concentrations of triglycerides, total-, HDL- and LDL-cholesterol, ALT, GGT, HbA1c, glucose, insulin and C-peptide were determined by routine laboratory analyses.

### Metabolic assessment

Fasting serum concentrations of blood lipids (triglycerides, cholesterol, high-density lipoprotein (HDL) cholesterol, low-density lipoprotein (LDL) cholesterol), glucose, liver enzymes [alanine transaminase (ALT), gamma-glutamyl-transferase (GGT)] as well as hemoglobin A_1c_ (HbA_1c_) were determined by routine laboratory methods at the Department of Laboratory Medicine, Medical University of Vienna; insulin and C-peptide were measured by chemiluminescence immunoassays (Roche Diagnostics GmBH).

Insulin resistance was assessed by the homeostatic model assessment index of insulin resistance (HOMA-IR) calculated as the product of the fasting serum insulin concentration (in milliunits per liter) and fasting plasma glucose concentration (in millimoles per liter) divided by 22.5 [31] and incremental area under the curve (AUC) was calculated for the time course of glucose, insulin and C-peptide concentration during the OGTT. Post-load hypoglycemia was defined by a blood glucose concentration<60 mg/dl (3.3 mmol/l) in the 2^nd^ hour of the OGTT. We calculated beta cell function during the entire OGTT by calculating the dynamic area under the curve of serum C-peptide concentrations as total AUC-(180× basal concentration) [32]. Established OGTT-derived indexes such as the Matsuda Index, the insulinogenic index and the disposition index were calculated in order to assess insulin sensitivity and beta cell function [33]. Disposition index was calculated as insulinogenic index * Matsuda index. Insulinogenic index was calculated as [ΔInsulin30-0(microU/ml)/ΔGlucose30–0(mmol/l)*18]. The C-peptide AUC to insulin AUC ratio was calculated as a measure of hepatic insulin extraction.

Metabolic syndrome was assessed according to the of International Diabetes Federation (IDF) consensus criteria published in 2006 [34], i.e. central obesity (waist >80 cm in women and >94 cm in men), plus any two of the following criteria: raised triglycerides (≥150 mg/dl), reduced HDL-cholesterol (<50 mg/dl in women and <40 mg/dl in men), raised blood pressure (systolic ≥130 mmHg or diastolic ≥85 mmHg or treatment for previous diagnosed hypertension) or raised fasting plasma glucose (≥100 mg/dl or previously diagnosed diabetes mellitus type 2).

Serum IGF-1 concentration pre- and one year post surgery was determined by chemiluminescent immunoassay on a Siemens Immulite analyzer (Siemens Healthcare Medical Diagnostics, Bad Nauheim, Germany). The assays were performed according to the manufacturer's recommendations by skilled technical laboratory personal methods at the Department of Laboratory Medicine, Medical University of Vienna. Interassay coefficients of variation varied between 2.0–8.5% and the lowest detection limit was 15 ng/ml.

The presence of fatty liver was calculated by an algorithm based on BMI (in kg/m^2^), waist circumference (cm), serum triglycerides (mg/dl) and GGT (U/l), called the “fatty liver index” (FLI), with an accuracy in detecting fatty liver of 0.84 (95%CI: 0.81–0.87) [35]. FLI is calculated as (*e* ^(0.953×loge (triglycerides)+0.139×BMI+0.718×loge(GGT)+0.053×waist-15.745)^/(1+*e* ^(0.953×loge (triglycerides)+0.139×BMI+0.718×loge(GGT)+0.053×waist-15.745)^)*100 [35].

### Statistical analysis

Statistical analysis included all patients. Continuous variables are presented as mean ± SEM if normally distributed, otherwise as median (IQR). Differences between continuous variables before and one year after bariatric surgery were analyzed by paired t-test or the non-parametric Wilcoxon signed rank test, as necessary. Categorical variables were compared by chi-square test. Differences between continuous variables in patients experiencing or not post-load hypoglycemia were calculated by unpaired student's t-test for normally distributed variables otherwise by Mann Whitney U-Test. Correlations were explored by Spearman's rank method. Odds ratios were computed by logistic regression to estimate the predictive value of IGF-1 on post-load hypoglycemia. The risk estimation model for post-load hypoglycemia was calculated for each participant using receiver operating characteristic (ROC) curves. The ROC-AUC and 95% CIs were estimated by non-parametric methods. All analyses were performed with IBM SPSS Statistics 20.0 (IBM Corporation, New York, USA). Differences were considered statistically significant with two sided p<0.05 and there were no considerations to adjust for multiplicity.

## Results

### Patients one year after bariatric surgery are at risk for post-load hypoglycemia

Post-operative changes in anthropometric and laboratory parameters were dramatic (Table 1). Patients on average lost 15.1±0.8 BMI units, corresponding to a mean weight loss of 43.2±2.5 kg. Parallel to the weight loss, we observed a significant reduction in the number of patients with metabolic syndrome, from 19 before surgery to 2 one year after (p<0.0001). Accordingly, we detected a significant improvement in patients' lipid profiles (triglycerides, total, LDL- and HDL-cholesterol, all p<0.01). Circulating concentration of liver enzymes ALT and GGT was also reduced, as was the fatty liver index (FLI) (all p<0.01), indicating a reduction of obesity-associated fatty liver disease.

**Table 1:** Characteristics of obese patients before and one year after bariatric surgery.

	pre-operative	post-operative	P
Sex (f/m)	29/7		
Age (y)	39.9±2.0	41.3±2.0	
Metabolic syndrome (IDF)^a^	19/36	2/36	<0.0001
***Anthropometric measurements***			
BMI (kg/m^2^)	45.4 (41.0–50.7)	30.9 (27.5–34.3)	**<0.0001**
Weight (kg)	130.2±3.7	87.3±2.9	**<0.0001**
Waist (cm)	131.0±2.2	102.2±2.5	<0.0001
WHR	0.92±0.01	0.88±0.01	**0.003**
Systolic BP (mmHg)	128±3	112±2	**0.0002**
Diastolic BP (mmHg)	83±2	71±2,	**0.0001**
***Metabolic Parameters***			
Triglycerides (mg/dl)	154±9	98.8±5.8	**<0.0001**
Total cholesterol (mg/dl)	205±5	165.5±5.3	**<0.0001**
HDL-C (mg/dl)	47±2	50.7±1.4	**0.002**
LDL-C (mg/dl)	127.8±4.9	95.0±4.2	**<0.0001**
ALT (U/l)	32±3	21.0±1.5	**0.003**
GGT (U/l)	39±4	19.7±1.8	**<0.0001**
FLI	96.9±0.5	51.9±5.7	**<0.0001**
HbA1c%	5.5±0.1	5.2±0.1	**<0.0001**
Fasting Glucose (mg/dl)	94±2	78±1	**<0.0001**
Insulin (µU/ml)	17.3 (7.4–28.8)	2.0 (2.0–5.4)	**<0.0001**
C-peptide (ng/ml)	4.0±0.3	1.8±0.1	**<0.0001**
AUC glucose (mg/dl*2 h)	140.6±4.1	77.4±6.6	**<0.0001**
AUC insulin (µU/ml*2 h)	117.8±21.8	79.2±10.3	**0.019**
AUC C-peptide (ng/ml*2 h)	11.5±0.8	10.8±0.7	0.31
HOMA-IR	3.9 (1.6–6.8)	0.4 (0.4–1.0)	**<0.0001**
IGF-1 (ng/ml)	125.1±8.2	145.6±11.5	0.053

a Metabolic syndrome was defined according to IDF criteria;

ALT, alanine transaminase; BP, blood pressure; FLI, fatty liver index; GGT, gamma-glutamyl-transferase; HDL-C, high density lipoprotein cholesterol; LDL-C, low density lipoprotein cholesterol, WHR, waist to hip ratio.

**Figure 1:**
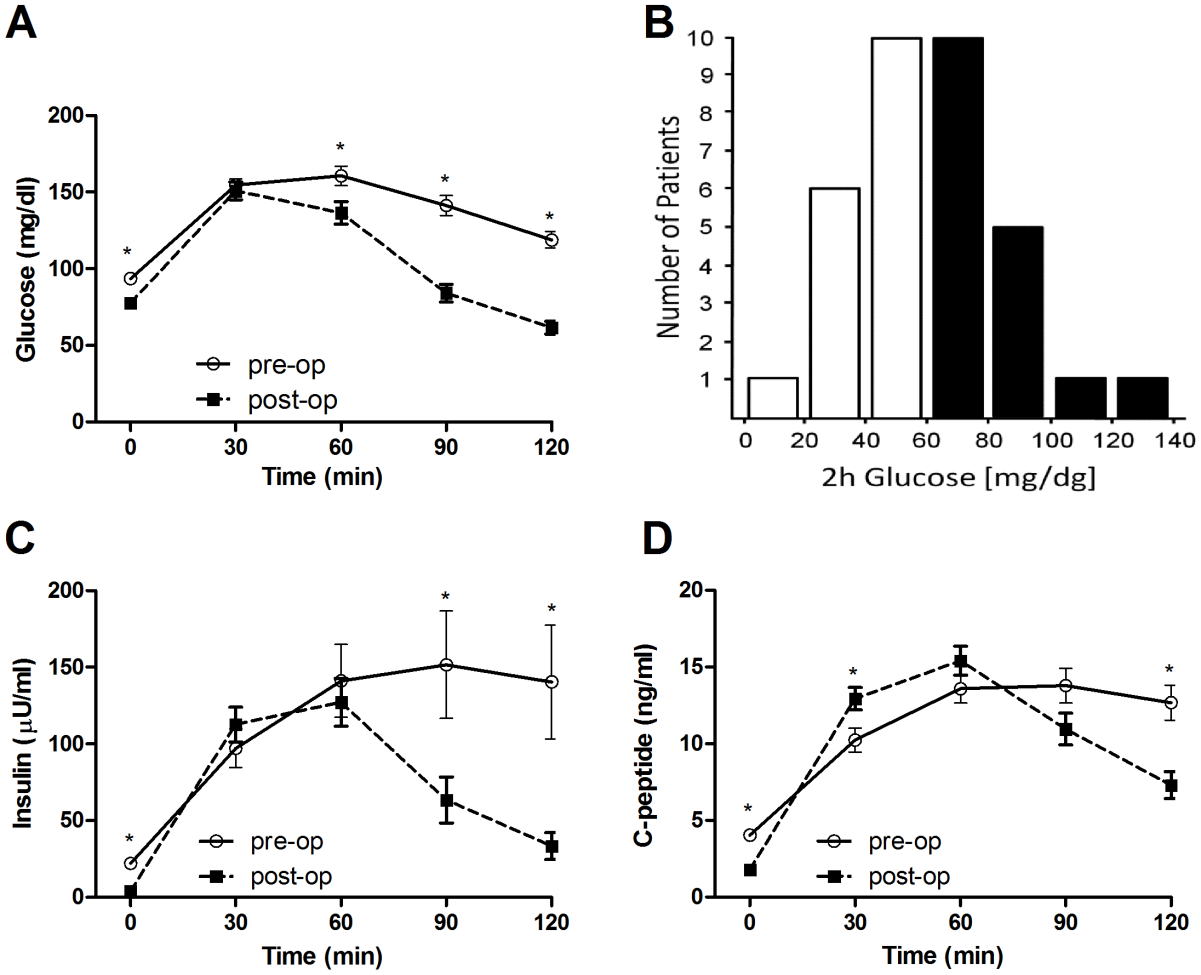
Glucose, insulin and C-peptide concentration during pre-operative and post-operative OGTT. (A) Glucose concentrations during the course of the pre-operative 2 h OGTT (n = 35, full lines) compared to the post-operative OGTT (n = 35, dashed lines). (B) Histogram of post-operative 2 h glucose concentrations during the OGTT (n = 35). Post-load hypoglycemia was defined by a 2 h glucose concentration<60 mg/dl. (C) Insulin and (D) C-peptide concentrations during the course of the pre-operative 2 h OGTT (n = 35, full lines) compared to the post-operative OGTT (n = 35, dashed lines).

Plasma HbA_1c_ concentration after surgery was lower than before surgery and in the normal range at both time-points. Fasting plasma glucose and fasting serum insulin concentrations were significantly reduced after the surgery, as was HOMA-IR (all p<0.0001). Glucose concentration during the course of the 2 h-OGTT was drastically changed in the postoperative setting, with significantly lower concentrations after the first hour, which is also reflected by the reduced area under the curve (AUC) - Figure 1A, Table 1. Moreover, post-load hypoglycemia as defined by blood plasma glucose concentration <60 mg/dl at 2 h of the OGTT was detected in half (50%) of all patients (n = 18, Figure 1B). These were mainly patients who underwent RYGB (n = 16). Surprisingly, most patients who experienced post-load hypoglycemia reported no complaints at all, including one patient whose blood glucose concentration reached 14 mg/dl (0.78 mmol/l). First phase insulin response to glucose load (calculated as the insulinogenic index) remained similar to the response observed before surgery, but insulin levels were significantly reduced during the second hour of the OGTT (Figure 1C). The post-operative AUC for insulin concentration was significantly lower than before surgery (p = 0.019; Table 1). Interestingly, the C-peptide concentration at 30 minutes of the test was higher after compared to before the operation (Figure 1D; p = 0.001), but the AUC for C-peptide concentration was not significantly different (Table 1). Serum IGF-1 concentration increased after surgery by a mean of 20.5±10.2 ng/dl and the increase was significant in trend (p = 0.053).

### Characteristics of patients with post-load hypoglycemia

**Figure 2:**
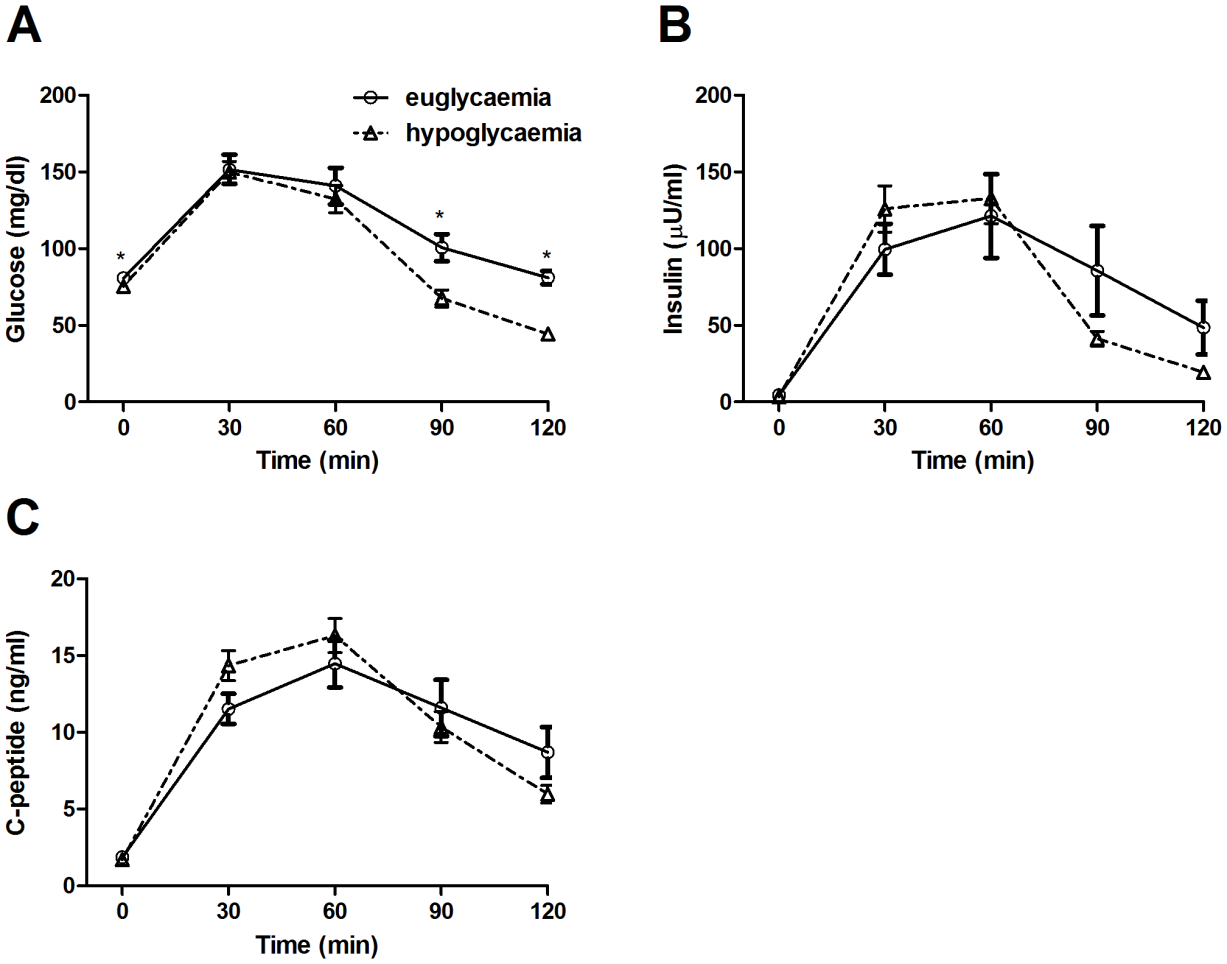
Post-operative OGTT glucose, insulin and C-peptide concentration in patients with euglycemia and hypoglycemia. (A) Glucose, (B) insulin and (C) C-peptide concentrations during the course of the post-operative 2 h OGTT in patients with euglycemia (n = 17, full lines) and patients with post-load hypoglycemia (n = 18, dashed lines). Differences between pre and post-operative values were calculated with paired t-test. Differences between patients with euglycemia and hypoglycemia were calculated by unpaired student's t-test.*p<0.05.

We split patients into two groups according to their glycaemic condition at 2 h during the post-operative OGTT (post-load hypoglycemia/euglycemia) and compared the changes in glucose, insulin and C-peptide concentrations and respective AUC. We detected significant differences in glucose concentration at time point 0′, 90′ and 120′ during the OGTT (p = 0.020, p = 0.003 and p<0.0001 respectively, Figure 2A), but insulin or C-peptide concentrations during the course of the OGTT were not altered (Figure 2B,C) except for a slight elevation of C-peptide concentration at 30 minutes (p = 0.06). The calculated AUC for glucose, insulin and C-peptide were similar in both groups (Table 2). Beta cell function as assessed by the dynamic AUC of serum C-peptide was not significantly different between the two groups (p = 0.47). There was no difference in the established OGTT-derived indices: Matsuda index, insulinogenic index and disposition index between the two groups (data not shown). Hepatic insulin extraction as calculated by the C-peptide AUC to insulin AUC ratio was not different between the groups (p = 0.24) and was not related to IGF-1 levels before or after surgery.

**Table 2:** Characteristics of patients with post-load hypoglycemia and euglycemia at 2 h during the post-operative OGTT.

	post-load hypoglycemia	euglycemia	P
Age	36.3±2.6	44.7±2.8	**0.037**
***Anthropometric measurements***			
BMI (kg/m^2^)	31.1 (27.0–35.9)	30.8 (28.0–34.7)	0.88
Weight (kg)	87.0±4.8	88.0±3.6	0.87
Waist (cm)	99.3±3.5	105.6±3.6	0.22
WHR	0.85±0.01	0.91±0.02	**0.018**
***Metabolic Parameters***			
Triglycerides (mg/dl)	84.1±5.0	114.6±9.7	**0.010**
Total cholesterol (mg/dl)	160.4±6.2	171.2±9.0	0.33
HDL-C (mg/dl)	51.1±2.3	49.9±1.8	0.69
LDL-C (mg/dl)	92.5±5.1	98.4±7.2	0.51
ALT (U/l)	20.4±2.3	20.7±1.9	0.93
GGT (U/l)	17.1±2.3	23.1±2.8	0.10
FLI	46.2±7.8	61.3±8.7	0.21
HbA1c %	5.2±0.1	5.3±0.1	0.34
Fasting Glucose (mg/dl)	75±2	81±1	**0.019**
Insulin (µU/ml)	2.0 (2.0–5.2)	2.0 (2.0–5.4)	0.88
C-peptide (ng/ml)	1.7±0.1	1.9±0.2	0.49
AUC glucose (mg/dl*2 h)	78.8±7.2	75.9±11.4	0.84
AUC insulin (µU/ml*2 h)	75.5±8.4	83.2±19.6	0.72
AUC C-peptide (ng/ml*2 h)	10.9±0.8	10.7±1.2	0.92
HOMA-IR	0.4 (0.4–1.0)	0.4 (0.4–1.1)	0.37
IGF-1 (ng/ml)	168.3±17.1	112.8±8.9	**0.007**

ALT, alanine transaminase; FLI, fatty liver index; GGT, gamma-glutamyl-transferase; HDL-C, high density lipoprotein cholesterol; LDL-C, low density lipoprotein cholesterol; WHR, waist to hip ratio.

By comparing anthropometric and laboratory parameters of patients with post-load hypoglycemia and patients with euglycemia, we found that age, WHR, serum triglyceride, fasting plasma glucose and IGF-1 serum concentrations were lower in patients with post-load hypoglycemia - Table 2. Glucose concentration at 2 h during the OGTT correlated with post-operative WHR (rho = 0.48, p = 0.004) and serum triglyceride concentration (rho = 0.50; p = 0.002). Other parameters, including BMI, serum lipids other than triglycerides, liver enzymes and FLI, did not differ between the groups.

Differences between the two groups in baseline anthropometric and laboratory parameters before surgery are presented in Table S1.

### IGF-1 serum concentration before and after surgery

**Figure 3:**
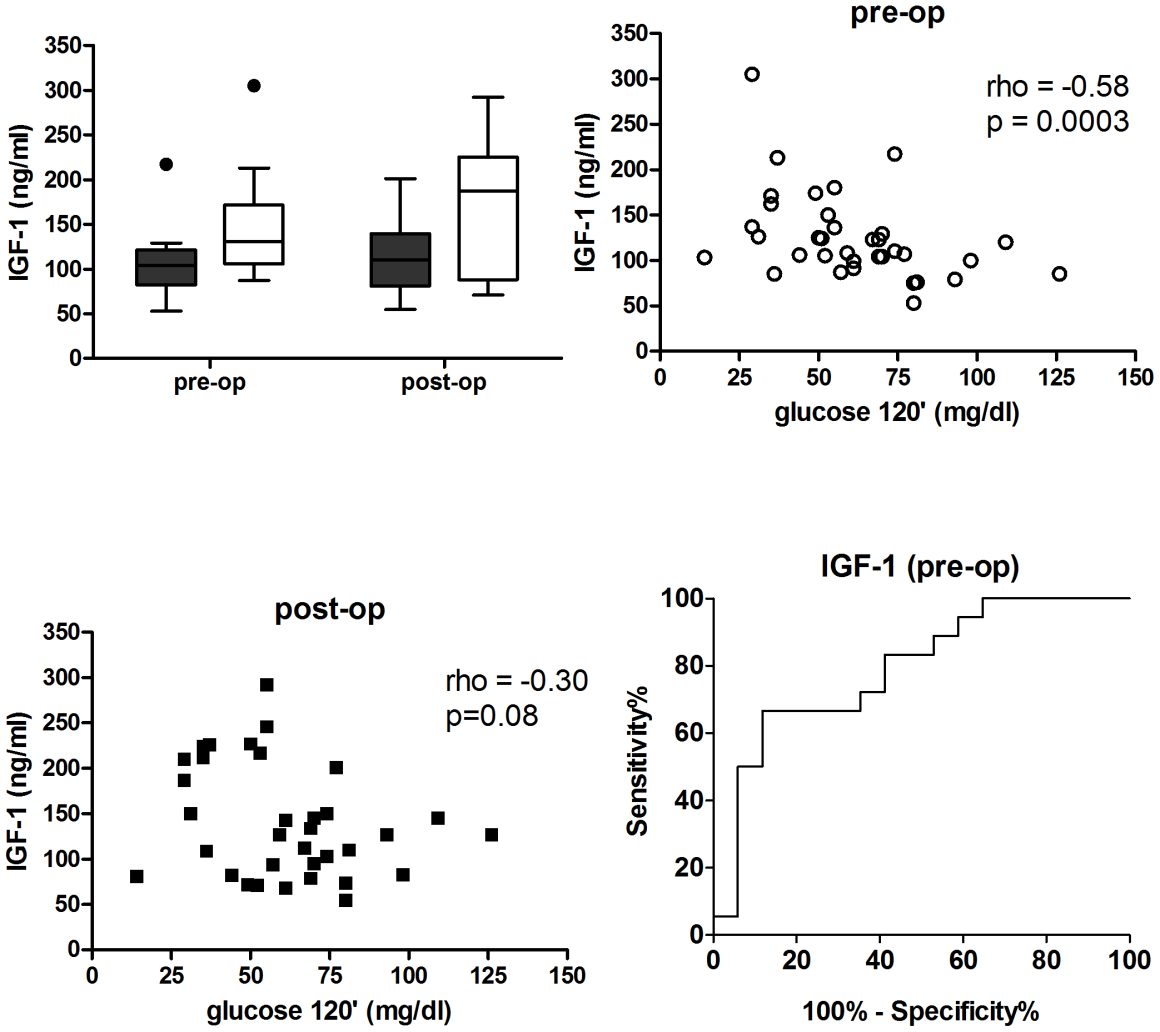
IGF-1 in patients with euglycemia and post-load hypoglycemia at 2 h during the post-operative OGTT. (A) Box-plots of pre- and post-operative serum IGF-1 concentrations in patients with euglycemia (n = 17, black bars) and post-load hypoglycemia (n = 18, white bars). Outliers are represented by dots. (B) Pre-operative serum IGF-1 concentration plotted against 2 h glucose concentration during the post-operative OGTT (n = 35). (C) Post-operative serum IGF-1 concentration plotted against 2 h glucose concentration during the post-operative OGTT (n = 35). (D) ROC-AUC curve for detecting post-load hypoglycemia according to pre-operative IGF-1 concentration (n = 35). Differences between IGF-1 concentrations between the two groups were calculated by unpaired student's t-test. The association between pre- and post-operative serum IGF-1 concentration and post-operative 2 h glucose concentration during the OGTT was analyzed using Spearman's rank correlation,*p<0.05.

We hypothesised that increased IGF-1 concentration could contribute to the susceptibility to develop post-load hypoglycemia. As shown in Table 2, mean post-operative IGF-1 serum concentration for all patients increased in trend, compared to IGF-1 concentration measured before the operation (146±12 vs. 125±8 ng/ml; p = 0.0535). Notably, post-operative IGF-1 concentration was significantly higher in patients with post-load hypoglycemia (n = 18) compared to patients with euglycemia (n = 17, Figure 3A). Interestingly, patients with post-load hypoglycemia had significantly higher serum IGF-1 concentrations compared to patients with euglycemia, already before surgery (145±12 ng/dl vs. 105±9 ng/dl respectively, p = 0.0122). When we analysed other differences in other pre-operative parameters with respect to post-load hypoglycemia, we found that only IGF-1 and age were significantly different between patients with hypoglycemia and euglycemia. Pre-operative IGF-1 serum concentration correlated negatively with glucose concentration at 2 h during the OGTT (rho = −0.58, p = 0.0003), whereas post-operative IGF-1 concentration correlated only in trend (rho = −0.30, p = 0.08; Figure 3B,C). The risk of post-load hypoglycemia one year after bariatric surgery increased by 28% with every 10 ng/ml increment of pre-operative IGF-1 serum concentration (OR 1.28; 95%CI: 1.03–1.55; p = 0.0297) and by 18% with post-operative IGF-1 concentrations (OR 1.18; 95%CI: 1.03–1.33; p = 0.0155). Moreover, patients with a pre-operative serum IGF-1 concentration above the calculated median of the whole cohort of 111 ng/ml had a significantly higher risk of post-load hypoglycemia (OR 4.8; 95%CI: 1.15–20.1; p = 0.0275) while the median cut-off value for post-operative IGF-1 concentration was not significantly associated with a higher risk. Multiple logistic regression for post-operative parameters including IGF-1, age, triglycerides and WHR revealed that only IGF-1 significantly predicted the occurrence of post-load hypoglycemia (OR 1.22; 95%CI: 1.02–1.42, p = 0.0300). The ROC analysis of the risk estimation by pre-operative IGF-1 revealed an AUC of 0.79 (95%CI: 0.64–0.94, p = 0.0033) and hence showed a good accuracy to detect hypoglycemia one year after bariatric surgery (Figure 3D; ROC-AUC of 1.00 represents a perfect test and 0.50 an insignificant test).

## Discussion

The main finding of our study was that post-load hypoglycemia approximately one year after bariatric surgery was closely associated with increased circulating IGF-1 concentrations before and after surgery. This could indicate that IGF-1 plays an important role in glucose homeostasis following RYBG.

We noticed a trend increase in post-operative IGF-1 serum concentration compared to pre-operative values. This increase could be attributed to an improvement in liver function following weight loss. Indeed obesity-associated fatty liver disease is likely to improve after weight loss, as reflected by a reduction of BMI, WHR, serum triglycerides and GGT, i.e. the most relevant variables for determining the fatty liver index. However, the reduction of hepatic steatosis, reflected by a reduced fatty liver index, does not seem to affect the risk of hypoglycemia, since both hypoglycemic as well as euglycemic patients had similar fatty liver indices. Therefore, we believe that IGF-1 is in itself a good predictor of post-load hypoglycemia, irrespective of improvements in liver function.

Here we identified increased IGF-1 concentrations before and after surgery as a major risk factor for post-load hypoglycemia. IGF-1 is synthesized in the liver, has structural similarities to proinsulin [36] and augments insulin actions such as stimulating glucose uptake, via phosphorylation of IRS-1, and suppresses hepatic gluconeogenesis [37], [38]. Thus, IGF-1 is known to enhance insulin sensitivity in humans and recombinant IGF-1 induces hypoglycemia as does its ectopic secretion [30], [39], [40]. Interestingly, both pre- and post-operative IGF-1 serum concentrations were significantly higher in subjects with post-load hypoglycemia and the pre-operative levels correlated negatively with 2 h glucose concentration. Thus, IGF-1 concentration was related to hypoglycemia risk. Moreover pre-operative IGF-1 serum concentration was the only parameter to be detected before surgery that predicted post-load hypoglycemia. Post-operative IGF-1 serum concentration was also predictive for the risk of hypoglycemia, alone and even after adjustment for other potential risk factors such as age, WHR and triglycerides. Higher IGF-1 concentrations can be detected under physiologic conditions in the young and pathologically in patients with growth hormone secreting pituitary adenomas (acromegaly or gigantism), but these patients present with hyperglycemia. IGF-1 secretion decreases with increasing age and we also observed a negative correlation between pre and post-operative IGF-1 concentration and age (r = -0.53, p = 0.0008 and r = −0.41, p = 0.013, respectively). We found no association between IGF-1 and serum triglycerides or IGF-1 and WHR, similar to the findings of Gram et *al* [41]. Furthermore, triglycerides and WHR were no longer significant when added to a multiple logistic regression for hypoglycemia risk, indicating the independent relation of IGF-1 to hypoglycemia.

Patients suffering from hypoglycemia during the OGTT had a similar BMI, but patients with hypoglycemia were younger, had lower WHR, and lower serum triglyceride concentrations than patients with euglycemia. This is partly consistent with work suggesting that diabetes remission is more common in younger, leaner, more insulin sensitive patients with adequate beta-cell function at baseline [42]. These latter correlations suggest that reductions in abdominal fat may be important for decreasing blood lipids and alleviating lipotoxicity effects on post-prandial blood glucose regulation. A possible explanation for post-load hypoglycemia may have been differences in insulin concentrations [9], but we noticed similar insulin and C-peptide responses to the oral glucose load in patients with hypoglycemia and patients with euglycemia. Furthermore both groups were comparable with respect to circulating serum total-, LDL- and HDL-cholesterol, liver enzymes, hepatic insulin extraction and fatty liver index.

Since clinical predictors for post-operative hypoglycemia are missing, we aimed to identify potential risk factors for post-load hypoglycemia occurring one year after bariatric surgery, in a cohort of non-diabetic patients. Strikingly, in an unexpectedly high percentage, namely half of all patients, we detected moderate to severe post-load hypoglycemia at 2 h during an OGTT. This finding was surprising, as the frequency for hypoglycemia following RYGB described in the literature is much lower [1], [4], [7]–[9]. Emerging evidence of an increased susceptibility to postprandial hypoglycemia following gastric bypass surgery [43] points to the fact that hypoglycemia might contribute to the increased risk of death from non-disease related causes, such as accidental deaths and suicide [2], [44], as hypoglycemia is known to determine an increase in depressive symptoms [45]. Similar to patients under insulin treatment for type 1 or type 2 diabetes, most of the patients we evaluated were completely unaware of hypoglycemia during the test. Hence these patients could suffer from hypoglycemia-related impairments of cognitive and motor functions [46] without being aware of it. Therefore, we believe it is crucial to identify patients at risk for post-prandial hypoglycemia following bariatric surgery. We detected a clinically applicable “threshold” of 111 ng/ml in pre-operative IGF-1 concentration which can be somewhat useful in assessing the risk of hypoglycemia. The association of insulin resistance with IGF-1 concentrations seems to be U shaped [23], [24] in patients who did not undergo bariatric surgery and there is not enough data to make such an assumption for bariatric surgery patients. Nevertheless, higher values are predictive of post-load hypoglycaemia following bariatric surgery. Of note, serum IGF-1 concentration can be determined independent of an elaborate and time-consuming oral tolerance test, which further requires patient compliance. As an additional advantage, IGF-1 concentration as a predictive marker can be determined even before the operation and may hence contribute to patient selection for gastric bypass surgery, while all other parameters relating to post-operative hypoglycemia – except from age – need to be determined in the post-operative setting, thus demanding a strict follow-up of patients.

We are aware of some limitation to our study, including the small sample size and the fact that we could not compare the incidence of post-load hypoglycemia across a wider spectrum of surgical techniques, since the most prevalent surgical procedure performed in this cohort, was RYGB. Although the sample size was modest, the differences we observed were consistent enough to encourage further researcher. As for the operating technique, RYGB is most representative, since it is the most commonly performed bariatric procedure worldwide [47]. Moreover, 80% of the patients were women, which is a common finding in bariatric surgery literature and we cannot make the assumption our findings would be identical in men. Further work should be performed including more men and also type two diabetics, since we included only non-diabetic patients.

In conclusion, post- and particularly pre-operative IGF-1 serum concentrations help in identifying patients at risk for developing post-load hypoglycemia. Identification of patients at hypoglycemia risk based on IGF-1 may improve patient selection for gastric bypass surgery, help to tailor dietary recommendations, promote awareness of patients and health-care professionals for this complication, and may hence further improve the prognosis of these patients.

## Supporting Information

Table S1 **Baseline characteristics (before surgery) of patients who underwent post-load hypoglycemia and euglycemia at 2h during the post-operative OGTT.**
(DOCX)
