# Supplementary material for: Insulin-Like Growth Factor 1 Predicts Post-Load Hypoglycemia following Bariatric Surgery: A Prospective Cohort Study
Source: PLoS One. 2014 Apr 15;9(4):e94613. doi: 10.1371/journal.pone.0094613 (PMC3988194; doi:10.1371/journal.pone.0094613)
Supplement: Table S1 — Baseline characteristics (before surgery) of patients who underwent post-load hypoglycemia and euglycemia at 2h during the post-operative OGTT. (DOCX) [file pone.0094613.s001.docx]

**Table S1 – Baseline characteristics (before surgery) of patients who underwent post-load hypoglycemia and euglycemia at 2h during the post-operative OGTT**

|  | | post-load  hypoglycemia | | euglycemia | P |
| --- | --- | --- | --- | --- | --- |
| Age | 36 ± 3 | | 45 ± 3 | | **0.0370** |
| *Anthropometric measurements* |  | |  | |  |
| BMI (kg/m^2^) | | 49.6 (43.1 – 51.5) | | 42.7 (40.4 – 46.8) | 0.05 |
| Weight (kg) | | 135.9 ± 5.9 | | 125.2 ± 4.6 | 0.16 |
| Waist (cm) | | 132.9 ± 3.5 | | 129.9 ± 2.7 | 0.50 |
| WHR | | 0.89 ± 0.01 | | 0.94 ± 0.03 | 0.07 |
| *Metabolic Parameters* | |  | |  |  |
| Triglycerides (mg/dl) | | 146 ± 12 | | 165 ± 13 | 0.30 |
| Total cholesterol (mg/dl) | | 203 ± 7 | | 208 ± 8 | 0.64 |
| HDL-C (mg/dl) | | 46 ± 3 | | 47 ± 2 | 0.63 |
| LDL-C (mg/dl) | | 128.2 ± 8.8 | | 127.9 ± 7.7 | 0.97 |
| ALT (U/l) | | 33 ± 5 | | 32 ± 4 | 0.84 |
| GGT (U/l) | | 32 ± 4 | | 47 ± 6 | **0.048** |
| FLI | | 96.9 ± 0.9 | | 97.6 ± 0.3 | 0.44 |
| HbA1c % | | 5.5 ± 0.1 | | 5.6 ± 0.1 | 0.49 |
| Fasting Glucose (mg/dl) | | 94 ± 3 | | 94 ± 2 | 0.91 |
| Insulin (µU/ml) | | 19.5 (8.5 – 29.4) | | 16.1 (6.1 – 24.7) | 0.78 |
| C-peptide (ng/ml) | | 4.0 ± 0.5 | | 4.1 ± 0.5 | 0.88 |
| AUC glucose (mg/dl*2h) | | 135.7 ± 6.5 | | 145.9 ± 5.3 | 0.23 |
| AUC insulin (µU/ml*2h) | | 98.7 ± 13.9 | | 137.6 ± 43.9 | 0.32 |
| AUC C-peptide (ng/ml*2h) | | 10.7 ± 0.9 | | 12.3 ± 1.4 | 0.40 |
| HOMA-IR | | 4.2 (1.8 – 7.6) | | 3.6 (1.5 – 5.7) | 0.82 |
| IGF-1 (ng/ml) | | 145 ± 12 | | 105 ± 9 | **0.012** |

ALT, alanine transaminase; FLI, fatty liver index; GGT, gamma-glutamyl-transferase; HDL-C, high density lipoprotein cholesterol; LDL-C, low density lipoprotein cholesterol.
